# Redesigned and chemically-modified hammerhead ribozymes with improved activity and serum stability

**DOI:** 10.1186/1472-6769-4-1

**Published:** 2004-12-09

**Authors:** Philip Hendry, Maxine J McCall, Tom S Stewart, Trevor J Lockett

**Affiliations:** 1 CSIRO Molecular Science, PO Box 184 North Ryde NSW 1670, Australia; 2 School of Biochemistry and Molecular Genetics, University of New South Wales, Sydney NSW 2052, Australia

## Abstract

**Background:**

Hammerhead ribozymes are RNA-based molecules which bind and cleave other RNAs specifically. As such they have potential as laboratory reagents, diagnostics and therapeutics. Despite having been extensively studied for 15 years or so, their wide application is hampered by their instability in biological media, and by the poor translation of cleavage studies on short substrates to long RNA molecules. This work describes a systematic study aimed at addressing these two issues.

**Results:**

A series of hammerhead ribozyme derivatives, varying in their hybridising arm length and size of helix II, were tested *in vitro *for cleavage of RNA derived from the carbamoyl phosphate synthetase II gene of *Plasmodium falciparum*. Against a 550-nt transcript the most efficient (t_1/2 _= 26 seconds) was a miniribozyme with helix II reduced to a single G-C base pair and with twelve nucleotides in each hybridising arm. Miniribozymes of this general design were targeted to three further sites, and they demonstrated exceptional cleavage activity. A series of chemically modified derivatives was prepared and examined for cleavage activity and stability in human serum. One derivative showed a 10^3^-fold increase in serum stability and a doubling in cleavage efficiency compared to the unmodified miniribozyme. A second was almost 10^4^-fold more stable and only 7-fold less active than the unmodified parent.

**Conclusion:**

Hammerhead ribozyme derivatives in which helix II is reduced to a single G-C base pair cleave long RNA substrates very efficiently *in vitro*. Using commonly available phosphoramidites and reagents, two patterns of nucleotide substitution in this derivative were identified which conferred both good cleavage activity against long RNA targets and good stability in human serum.

## Background

**Figure 1:**
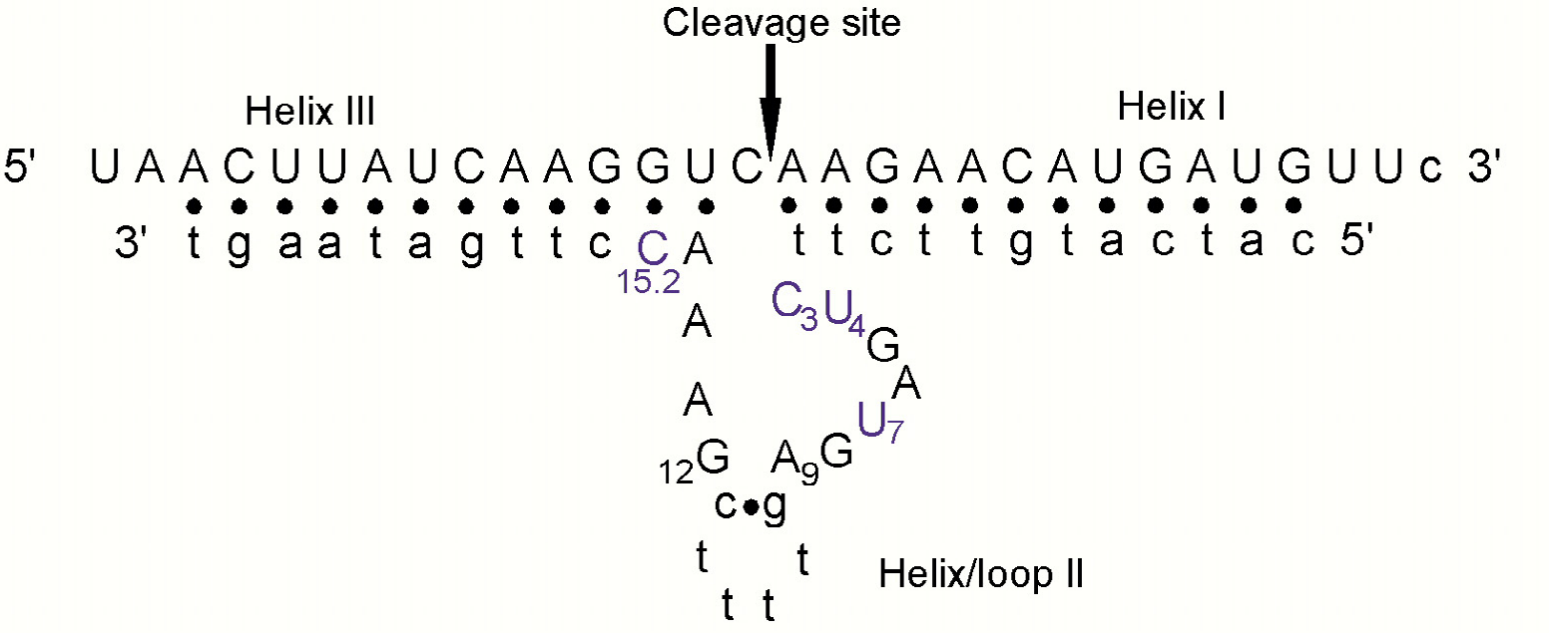
Schematic representation of Mrz-12/12-A bound to substrate S-30. Helices I and III are formed between the ribozyme and substrate. The standard hammerhead (eg Rz-12/12) has a 4 base pair helix II in place of the single g-c pair in the miniribozyme. The minizyme (Mz-12/12) has no helix II, but has a loop sequence gtttt connecting bases A_9 _and G_12_. Upper-case letters represent ribonucleotides, and lower-case letters represent deoxyribonucleotides. Nucleotides which have been further modified in this study are shown in blue.

Hammerhead ribozymes were discovered as self-cleaving motifs in a number of small, circular, pathogenic RNAs in plants [1-3]. Uhlenbeck [4] showed that the ribozyme was able to act in a bimolecular fashion as a true enzyme, *ie *each ribozyme was able to cleave multiple substrates. Haseloff and Gerlach [5] divided the hammerhead into a form in which the majority of the conserved nucleotides were located on the enzyme strand, with the only sequence requirements for the substrate being UH (H = A, U or C) [6-8]. Since 1988 this configuration, as shown in Figure 1, has been the paradigm for hammerhead ribozyme design. Hammerhead ribozymes are sequence-specific RNA cleaving agents with the potential to control the expression of genes by eliminating specific RNAs. This can be achieved by expressing the ribozyme within the target cell or by delivering it to the cell as a preformed entity. One of the difficulties associated with delivering preformed ribozymes is their instability *in vivo*, since RNA is degraded very rapidly by ribonucleases present in cells and extracellular fluids. Significant segments of ribonucleotides in the hammerhead ribozyme can be replaced with more nuclease-resistant analogues like DNA, phosphorothioate linkages, or 2' O-methyl analogues; however, within the conserved core of the hammerhead, the majority of ribonucleotides are sensitive to modification. Yang *et al *[9] demonstrated that predominantly DNA ribozyme analogues with at least 4 ribonucleotides (G_5_, G_8_, A_9 _and A_15.1 _or G_15.2 _numbered according to [10]) displayed measurable cleavage activity (albeit reduced 5000-fold). Phosphorothioate modification of DNA hybridising arms and three of the conserved pyrimidines (C_3_, U_4 _& U_7_) resulted in significant increase in stability in human serum with a 6-fold loss in cleavage activity [11]. In the context of 2'-O-methyl substituted ribozyme analogues, at least 5 unmodified ribonucleotides (G_5_, G_8_, A_9_, A_15.1 _and G_15.2_) were required for activity [9]. Paolella *et al *[12] identified a minimum set of 6 ribonucleotides, U_4_, G_5_, A_6_, G_8_, G_12 _and A_15.1_, in the conserved domain in which substitution with 2' O-allyl ribonucleotides inhibited activity. Eckstein's group [13,14] showed that good activity and stability in foetal calf serum could be achieved with 2'-amino-2'-deoxyuridines at U_4 _and U_7_, and 2'-fluoro-2'-deoxycytidine at C_3_. Hammerheads in which helix II was shortened to only two base pairs, and all the pyrimidines were 2'-fluoro or 2'-methoxyethoxy derivatives except for U_4 _and U_7 _which were 2'-amino-2'-deoxy, were only 2–3 fold less active than the unmodified parent hammerhead [15], but showed a 10^4^-fold increase in nuclease stability. A study of the effects of various modified nucleotides on stability and activity of the 2'-O-methyl-pyrimidine modified hammerhead found a number of modifications, including 2'-amino-2'-deoxyuridine, 2'-O-methyl-uridine, and 2'-C-allyl-uridine at positions U_4 _and U_7_, supported good rates of cleavage [16]. These modifications result in a greater than 10^3^-fold increase in stability in human serum, while the addition of an inverted thymidine at the 3' end of the oligonucleotide (a 3'-3' linkage) further improved the stability by two orders of magnitude.

Our laboratory is interested in the relationship between hammerhead design and reactivity. We have described a number of ribozyme derivatives that appear to have promise as RNA cleavage agents. Minizymes (Mz) possess a non-base-pairing tetranucleotide linker in place of helix II [17]. In general, such minizymes are less active than standard hammerheads, although in some instances they show comparable cleavage rate constants [18,19]. Miniribozymes (Mrz) have a single G_10.1_–C_11.1 _base pair joined by a flexible linker in place of helix-loop II [19]. Asymmetric hammerheads are those in which the 5' hybridising arm is restricted to around 5 or 6 nucleotides; this modification eliminates the decrease in cleavage rate that occurs with standard hammerheads when the length of helix I, formed upon binding the substrate, increases to greater than about 6 base pairs [20,21]. The purpose of this study was to investigate the ability of these various derivatives to cleave an RNA molecule (sequence derived from the mRNA of the cpsII gene of the Malaria-causing organism *Plasmodium falciparum *[22]) in the context of two substrates, a 30-mer and a 550-mer. Having optimised the cleavage activity of the ribozyme, we planned to chemically modify the nuclease sensitive RNA nucleotides, using readily available protected nucleoside phosphoramidites, to extend the life of the ribozyme in the presence of human serum.

## Results

### Ribozyme design

The primary target site for cleavage in this study was a previously identified site [23] in the cps II mRNA [22] of *Plasmodium falciparum*. The chosen target site, centred at position 3733 in the nucleotide sequence (Genbank reference L32150), has the local sequence 5' UAA CUU AUC AAG GUC* AAG AAC AUG AUG UUC 3', where the site of cleavage is denoted by the asterisk. A number of ribozyme designs were tested for their ability to cleave this RNA sequence either as a short (30-mer) oligonucleotide or in a transcribed RNA segment (550 nt). These designs included standard hammerhead ribozymes (Rz) which are defined as those with a helix II consisting of four base pairs closed at the end with a four-nucleotide loop, minizymes (Mz) [17] which lack helix II and instead the two segments of conserved nucleotides are linked between A9 and G12 with a non-base-paired linker (in this case consisting of 5 nucleotides), and finally miniribozymes (Mrz) [19] which have a single G-C base pair replacing helix II and in this instance a linker sequence consisting of four deoxythymidines. In this communication the core of each ribozyme is flanked by hybridising arms composed of DNA of various lengths, where the length is given in the ribozyme's name (e.g. Rz-6/12 has 6 nt in its 5' hybridising arm and 12 nt in its 3' arm). Hammerhead-ribozyme derivatives of these designs were tested for their ability to cleave the 30-mer substrate at pH 7.6, 37°C and 10 mM MgCl_2 _under pseudo first-order conditions with an excess of ribozyme. Ribozyme concentrations ranged from 50 nM to 10 μM. Cleavage data fitted well to single exponential curves to yield observed rate constants which were plotted against the ribozyme concentration in each experiment to determine the apparent dissociation constant ("K_d_") and the maximum cleavage rate constant (k_max_) for each ribozyme-substrate pair (Table 1). In terms of catalytic efficiency, the most efficient ribozyme was the standard hammerhead Rz-12/12. Its efficiency is due to a very low "K_d_" of 7 nM, despite displaying a k_max _some 5-fold less than Mrz-12/12. The highest k_max _was displayed by Rz-6/12; however it had a "K_d_" about 60-fold greater than Rz-12/12.

**Table 1:** Kinetic parameters for RNA/DNA unmodified hammerhead ribozyme derivatives.

**Ribozyme**	**S-30**			**S-550**		
	
	**k_max _(min^-1^)**	**"K_d_" nM**	**k_max_/"K_d_" (min^-1^μM^-1^)**	**k_max _(min^-1^)**	**"K_d_" nM**	**k_max_/"K_d_" (min^-1^μM^-1^)**
Rz-12/12	0.8 ± 0.1	7 ± 12	114 ± 200	0.7 ± 0.1	2600 ± 900	0.3 ± .2
Mz-12/12	0.22 ± 0.08	31 ± 6	7 ± 4	0.12 ± 0.01	240 ± 50	0.5 ± .2
Mrz-8/8	0.56 ± 0.06	210 ± 80	2.7 ± 1.3	0.005 ± 0.001	2200 ± 500	0.002 ± .001
Mrz-12/12	4.2 ± 0.3	75 ± 30	56 ± 26	1.6 ± 0.1	120 ± 50	13 ± 6
Rz-6/12	9 ± 1	400 ± 200	22 ± 13	0.6 ± 0.1	2800 ± 900	0.2 ± .1

Cleavage conditions; 37°C, pH 7.6, 10 mM MgCl_2_, ribozyme is in large excess over RNA substrates S-30 (30 nt) and S-550 (550 nt).

The abilities of all the ribozymes to cleave the same target in the context of transcribed RNA (550 nt) was also examined. Mrz-12/12, by virtue of only a modest decrease in k_max_, and marginal increase in "K_d_", was by far the most efficient of all the designs tested. In contrast Mrz-8/8, Rz-12/12 and Rz-6/12 displayed "K_d_"'s between 2 and 3 mM, which, in the case of Rz-12/12, is an increase of about 400-fold. Interestingly the k_max _values displayed by Rz-12/12 and Rz-6/12 were within experimental error, and were the same as displayed by Rz-12/12 for cleavage of the short substrate.

### Cleavage of other targets by the miniribozyme

We examined whether the effectiveness of the miniribozyme design was limited to this target site. Miniribozymes, with long (>10 nucleotides) hybridising arms were designed to cleave RNAs of interest to other projects in the laboratory. Tet Mrz was a 53-mer oligonucleotide with conserved bases of RNA and hybridising arms and stem loop II composed of DNA. This Mrz, with hybridising arms of 18 and 19 nucleotides, was targeted to cleave the GUC triplet at position 60 of the *Tetrahymena *IVS ribozyme [24]. It cleaved the 388-nt transcript, L-21 ScaI, with a rate constant of 4.0 ± 0.2 min^-1 ^(t_1/2 _= 10 seconds), to about 70%, at pH 7.6, 37°C and 10 mM MgCl_2_. Another miniribozyme with 14-mer arms (HC Mrz), targeting a segment of Hepatitis C polyprotein mRNA, was tested against a synthetic 29-mer substrate. Under our standard conditions about 70% of the substrate was cleaved, and the cleavage rate (>5 min^-1^) was too fast to be measured reliably. In contrast, the standard ribozyme HC Rz cleaved 78% of the same substrate with a rate constant of only 0.2 ± 0.02 min^-1^. Finally, PDGF MRz, with hybridising arms each of 10 ribonucleotides, was tested against both 25-mer synthetic and 707-nt transcribed substrates. Under standard conditions, the 25-mer was ~ 80% cleaved with a rate constant > 5 min^-1^. Reducing the magnesium ion concentration to 1 mM yielded a rate constant of 2.8 min^-1 ^and 63% cleavage for the 25-mer substrate, and 1.6 min^-1 ^and 45% cleavage for the 707-nt transcript.

### Identification of nuclease susceptible sites in human serum

**Figure 2:**
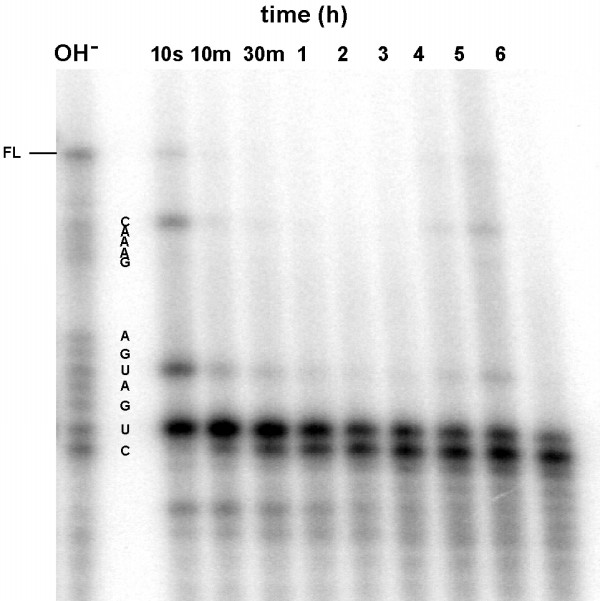
Degradation of 5' end-labelled Mrz-12/12 A in RPMI + 10% human serum at 37°C. Time of incubation in indicated above each lane. OH^-^indicates an alkaline digest of the same material. FL indicates the position of the full-length miniribozyme. The position of the fragments terminating at each of the ribonucleotides is indicated by the letters adjacent to the alkaline digest.

Mrz-12/12 was the most efficient cleaver of the 550-nt cpsII RNA transcript and was used as the platform to test the effect of chemical modification on cleavage activity and nuclease stability. Firstly, the stability of unprotected Mrz-12/12 in RPMI + 10% human serum was determined using ^32^P 5'-end labelled ribozyme at 37°C (Figure 2). Approximately 90% of the miniribozyme is degraded in 10 seconds, and no full-length material is observed after 10 minutes. Initially there are three main sites of cleavage, at U_4_, U_7 _and C_15.2_. After 10 minutes, nearly all the end-labelled material co-migrates with the band generated by alkaline digestion at U_4_. That initial, major product has a half-life of about two hours under these conditions, as it is slowly converted to a product one nucleotide shorter, *ie *its 3' end corresponds to C_3 _in the original miniribozyme.

### Nuclease resistant modifications

**Figure 3:**
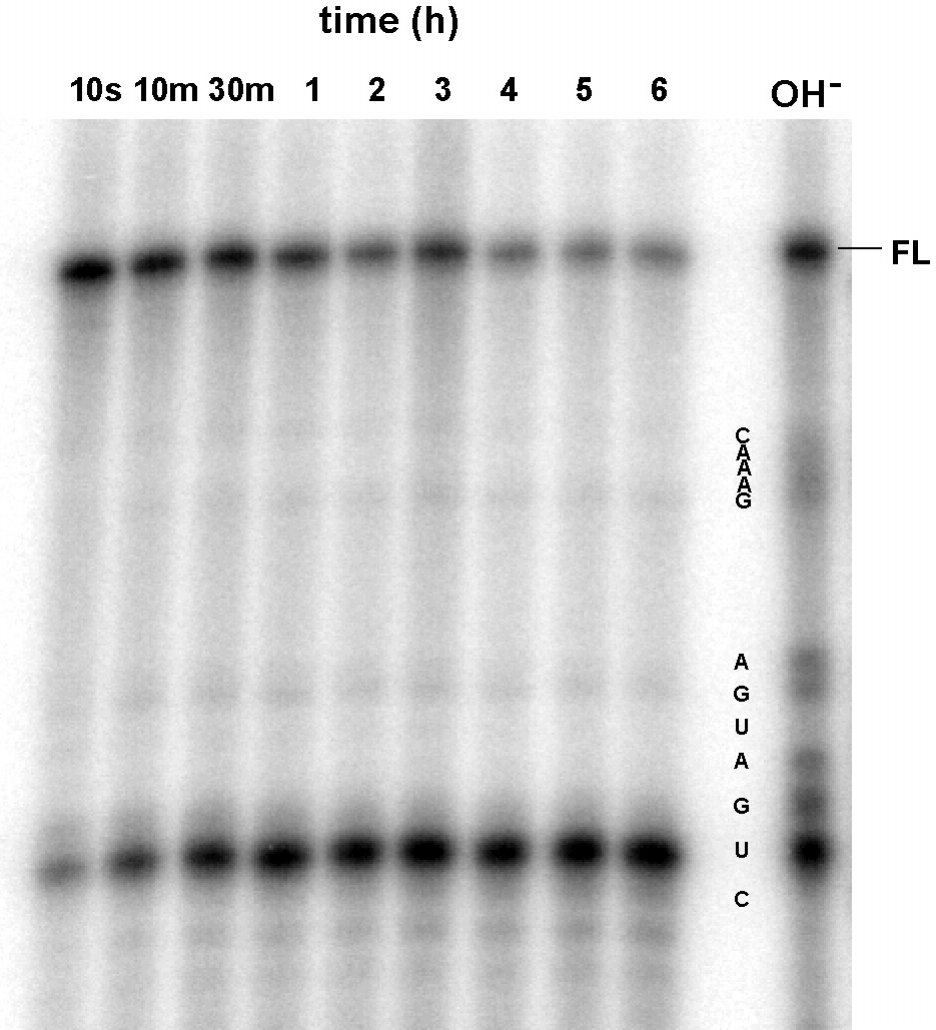
Degradation of Mrz-12/12 J in RPMI + 10% Human Serum. Experimental conditions are as described in Figure 2.

Commercially available modified phosphoramidites were used to generate hammerhead derivatives which were expected to be protected from nuclease degradation. The effect of the various modifications on the cleavage ability are given in Table 2. In these experiments the concentration of the miniribozyme was fixed at 1 μM and the substrate S-30 at 5 nM. The nucleotide most sensitive to chemical modification was U_4_. All the 2' modifications tested (amino, deoxythymidine, deoxyuridine, and O-methyl, shown as D, F, G and H, respectively, in Table 2) diminished activity by more than 10-fold. Only the presence of a phosphorothioate linkage between U_4 _and G_5 _preserved the activity of the unmodified ribozyme. The ready availability of 2'-O-methyl phosphoramidites, coupled with the previous demonstrations [9,16] of tolerance to that modification at C_3_, U_7 _and C_15.2_, lead us to synthesise Mrz-J, which we expected to have reasonable activity and nuclease stability. Its cleavage rate constant was actually twice that observed for the unmodified ribozyme, and its stability in serum was increased about 10^3^-fold. The kinetics of degradation in serum (Figure 3) were not straight-forward, displaying a rapid initial decay of about 25% of the starting material, followed by an approximately first-order decay with a half-life around 30 minutes. This second phase accounted for approximately 50% in the total starting material, *ie *after about 4 hours approximately 25% of the full-length material remained intact and thereafter decayed only very slowly. There was a single major product, corresponding to cleavage at U_4_, observed over the six hours of the experiment.

**Table 2:** Cleavage Rate constants for cleavage of S-30 by Chemically Modified Mrz-12/12.

**Miniribozyme (Mrz-12/12)**	**C_3_**	**U_4_**	**U_7_**	**C_15.2_**	**3' end**	**Cleavage rate constant (min^-1^)**	**Relative Stability**
A	-	-	-	-	-	4.2 ± 0.3	1
B	F	NH_2_	NH_2_	F	-	0.30 ± 0.05	-
C	-	NH_2_	NH_2_	-	-	0.18 ± 0.01	-
D	-	NH_2_	-	-	-	0.30 ± 0.03	-
E	-	-	NH_2_	-	-	7.7 ± 0.8	-
F	-	dT	-	-	-	0.014 ± 0.005	-
G	-	dU	-	-	-	0.024 ± 0.007	-
H	-	OMe	-	-	-	0.01 ± 0.007	-
I	-	ps	-	-	-	3.9 ± 0.06	-
J	OMe	ps	OMe	OMe	psps	7.3 ± 0.7	1400
K	OMe	NH_2_	OMe	OMe	-	0.6 ± 0.1	8600
Rz-12/12-L	-	NH_2_	-	-	-	0.05 ± 0.009	-

Cleavage conditions; 37°C, pH 7.6, 10 mM MgCl_2. _[Rz] = 1 μM, [S30] = 5 nM. (- = unmodified, ie 2'OH). F = 2'-fluoro, NH_2 _= 2'-amino, dT = 2'-deoxythymidine, dU = 2'-deoxyuridine, OMe = 2'-O-methyl, ps = 3' phosphorothioate linkage. Stability is defined as the time required in 10% human serum to degrade 75% of full-length ribozyme, relative to unmodified miniribozyme A.

**Figure 4:**
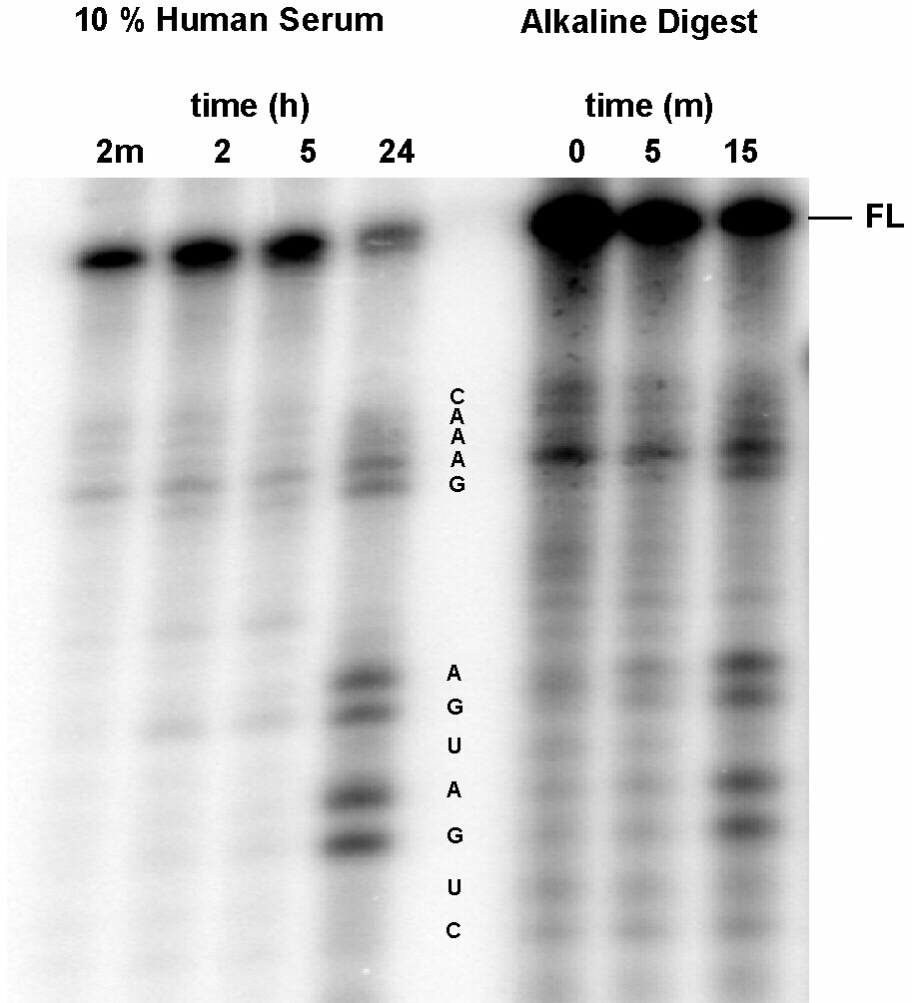
Degradation of Mrz-12/12 K in RPMI + 10% Human Serum, and by alkaline digest. Experimental conditions are as described in Figure 2.

The single degradation site in Mrz-J suggested that a more robust modification at this position would have a significant effect on its lifetime in serum. Mrz-12/12 K was synthesised with a 2'-amino modification at the U_4 _position and with a 2'-O-methyl modification at each of the three other conserved pyrimidines. Protection of the 3' end was omitted from Mrz-12/12 K because it did not appear to contribute significantly to the stability observed in Mrz-12/12 J. As expected, the cleavage activity of Mrz-12/12 K was significantly reduced compared to Mrz-12/12 J (Table 2), but stability in serum was greatly improved with only minor losses apparent after 5 hours incubation at 37°C (Figure 4). Even after 24 hours in 10% serum, approximately 25% of the radioactivity co-migrated with the full-length material, representing an almost 10^4^-fold increase in stability. Even in the absence of 3' terminal protection there was no 3' exonuclease activity apparent. The amount of ^32^P label appearing in the gel lanes remained relatively constant throughout the experiment implying a lack of significant phosphatase activity in the serum. The main sites for degradation were the remaining unprotected (purine) ribonucleotides.

The modifications to the conserved nucleotides in this study were all made in the context of a miniribozyme. Compared to a standard hammerhead, the catalytic domain is expected to be more conformationally flexible, and therefore it should not be assumed that all the changes described here can be directly applied to standard hammerheads with a helix II of four base-pairs. However, as for the miniribozyme, a standard ribozyme containing a 2'-amino modification at U_4_, was about 15-fold less efficient at cleaving S-30, (k_obs _= 0.05 min^-1^, Rz-12/12-L, Table 2), compared to the unmodified ribozyme (Rz-12/12).

## Discussion

Work in this laboratory [20,21] and elsewhere [25] has demonstrated that the cleavage kinetics of any conventional hammerhead ribozyme are significantly inhibited when the length of helix I exceeds about 6 bp. This has been ascribed to an interaction between helices I and II which stabilises an inactive conformation [20]. The angle between helices I and II changes with metal ion concentration [26], and we postulated that a similar change was required for the transformation between more and less active conformations of the ribozyme [20]. This general model is supported by more recent observations using a variety of techniques [27-31] which conclude that the dominant ground-state conformation of the hammerhead is inactive and is in equilibrium with the active form. The results of the present study are in accord with these conclusions.

It is commonly observed, for conventional hammerhead ribozymes, that cleavage efficiencies for long transcripts are about 2 orders of magnitude lower than for short substrates [32]. This has been observed here also for ribozyme derivatives of both the conventional design and of the short-armed (8-nt) miniribozyme (Table 1). In these cases the cause appears to reside largely in the apparent dissociation constant "K_d_". In contrast, the longer-armed miniribozyme (Mrz-12/12) shows excellent cleavage kinetics against both short and long substrates. It appears that the more flexible miniribozyme is better suited to binding to the target in the context of a long RNA. These data can be interpreted according to a simple model in which ribozymes possessing a stable helix II form a more rigid three-dimensional structure [33,34] which does not bind strongly to the long, folded substrate. When binding is achieved at very high ribozyme concentrations, the ribozyme-substrate complex is sterically hindered to such an extent that the complex is locked into a poorly active conformation. Hence Rz-6/12 and Rz-12/12 display similar maximal rate constants. In contrast, the Mrz lacking helix II is more flexible and readily adapts to binding the folded substrate with relatively minor effects on k_obs _and "K_d_". This observation is not specific to target or cleavage triplet, since four unrelated targets, including three long transcripts, one with an AUC cleavage triplet, were cleaved with rate constants much higher than typically reported for cleavage even of short substrates. It is worth noting the magnitude of the observed cleavage rate constants; under our standard conditions these miniribozymes cleave their targets with half-lives in the range of < 5 to 25 seconds.

The unmodified Mrz-12/12 was very unstable in 10% human serum. Degradation occurred by endonucleolytic cleavage at the 3' side of pyrimidine nucleotides. Since the hybridising arms and helix-loop II are composed of DNA, the four remaining ribo-pyrimidine nucleotides in the conserved domain were the critical residues for stabilisation. Modification of all four ribopyrimidines with 2'-aminouridine and 2'-fluorocytidine, (Mrz-12/12 B), resulted in a more than 10-fold decrease in maximum cleavage rate constant. This contrasts with some previous results where a U_4_, U_7_-amino derivatised ribozyme with a standard helix II was only marginally diminished in activity [14], and a U_4_, U_7_-amino derivatised ribozyme [16], in which all the remaining pyrimidines were modified with 2'-O-methyl groups, was only two-fold less active than the parent ribozyme. The study by Heidenreich *et al *[14] was confounded by the fact that the cleavage kinetics were measured against a long transcript and are relatively slow compared with rates typically observed for short substrates, and therefore it seems likely that access to the transcript, rather than chemistry of cleavage, may have been rate determining in that case. In a later report [15], a modification pattern identical to miniribozyme B (*ie *C_3_, C_15.2_-fluoro and U_4_, U_7_-amino) but in a ribozyme with a standard helix II resulted in a more than 10-fold loss in turnover number (k_cat_). The series of singly and doubly substituted miniribozymes (C-E) showed clearly that a 2'-aminouridine at position 4 was solely responsible for the loss in activity. This is consistent with the result of Beigelman *et al *[16], but, in contrast to that result, the cleavage activity of the miniribozyme was not rescued by the addition of an amino group at U_7_. A phosphorothioate linkage between U_4 _and G_5 _(Mrz-12/12 I) was the only modification tested here that did not suppress cleavage activity. This is in accord with interference studies [35,36] identifying U_4 _as insensitive to phosphorothioate modification. The results for dU and 2'O-methyl substitutions contrast with Beigelman *et al *[16] where, in a background of 2'O-methyl-pyrimidines, the effect of those substitutions at U_4 _were relatively minor.

In serum, Mrz-12/12 J was degraded by cleavage at the U_4 _phosphorothioate, where an initial, quite rapid degradation was followed by a slower step which is not complete at 6 hours. Chemically synthesised phosphorothioate linkages are a mixture of two enantiomers and it is likely that the multiphase kinetics observed were due in part to the different susceptibility of the two isomers (Rp and Sp) to the nucleases present in the serum [37,38]. Mrz12/12 K has 2'-amino modification at U_4_, is very stable in serum, and is only 7-fold less active than the unmodified parent. The products of nuclease cleavage of Mrz-12/12 K indicate that degradation occurred by cleavage at the unprotected ribopurine sites. This derivative was not protected at the 3' terminus, but unlike earlier work [11,16,38], there was no evidence in this experiment for 3'-DNA exonuclease activity.

In recent times RNAi has surplanted catalytic RNA as the method of choice for suppression of gene expression in many eukaryotic cells [39]. Results published to date suggest that it may be effective in many settings, including possibly as a therapeutic. However, the mechanistic details of RNAi activity are quite complex and are still being unravelled, and it seems likely that there will be cell types, perhaps whole classes of organisms, that lack the necessary cellular machinery. For this reason, it may be premature to abandon the autonomous catalytic RNAs. During the 1990's, many studies aiming to demonstrate hammerhead ribozymes as gene silencing agents *in vivo *were not successful. The results obtained here, and in other recent studies on the effect of non-conserved sequences on the kinetics of cleavage [25,40,41], are beginning to reveal shortcomings of many studies of that era. That is the *in vivo *catalytic efficiency of the hammerheads under study was compromised by their simplistic design, which was based on *in vitro *studies performed with very short substrates. We were too quick to ascribe poor cleavage of transcripts to "accessibility" issues, and too slow to undertake comprehensive studies to understand the phenomenon. The miniribozyme design, and the effective nuclease protection afforded by the simple chemical modifications described here, provide an opportunity to revisit cellular and *in vivo *experiments with hammerheads that are around two orders of magnitude more efficient.

## Conclusions

Long RNA substrates are much more effectively cleaved by miniribozymes than similarly targeted standard hammerheads. The presence of a stable helix II in the standard hammerhead appears to inhibit binding of the ribozyme to the substrate, and prevent the conformational mobility required for activity. Miniribozymes are affected by serum nucleases in a similar way to standard hammerheads, with cleavage occurring at the 3' side of unprotected ribopyrimidines. This work shows that it is readily possible to modify RNA/DNA hammerhead ribozymes, using commercially available reagents, to yield greatly improved nuclease resistance and retention of significant cleavage activity. These modified miniribozymes represent excellent candidates for cellular and *in vivo *studies on suppression of gene expression.

## Methods

### Oligonucleotide synthesis

Oligoribonucleotides were synthesised using DNA phosphoramidites and ancillary reagents supplied by Perkin Elmer (Applied Biosystems Division, Foster City, CA), RNA and modified phosphoramidites from Glen Research (Sterling, VA) and using an Applied Biosystems Model 394 DNA synthesiser. Phosphorothioates were introduced by oxidation with Beaucage reagent (Glen Research, Sterling, VA). Deprotection and purification of oligonucleotides were as described previously [20]. The purity of each oligonucleotide was checked by labelling its 5'-end with ^32^P phosphate using T4 polynucleotide kinase (New England Biolabs, Beverly, MA, USA) and [γ-^32^P]-ATP (Du Pont, Wilmington, DE), followed by electrophoresis in a 15% polyacrylamide gel containing 7 M urea and visualisation using a Molecular Dynamics PhosphorImaging system (Sunnyvale, CA). The concentrations of the purified oligonucleotides were determined by UV spectroscopy using the following molar extinction coefficients for the various nucleotides at 260 nm: A, 15.4 × 10^3^; G, 11.7 × 10^3^; C, 7.3 × 10^3^; T/U, 8.8 × 10^3 ^l mol^-1 ^cm^-1^. All oligonucleotides were stored in distilled, deionised and autoclaved water at -20°C.

The 550-nt CPSII substrate was transcribed by T7 RNA polymerase from the vector pCPS3b.1 [22] after linearisation with Eco RI restriction enzyme using an Ampliscribe T7 kit (Epicentre Technologies, Madison, WI, USA). The transcription reaction contained CTP, GTP and ATP (unlabelled) at 5 mM, UTP at 0.5 mM and ^32^P UTP at about 1 nM. The transcription was for 90 minutes at 37°C, followed by the addition of 0.5 μL of DNAaseI and incubation for a further 20 minutes. The mixture was extracted three times with a 1:1 phenol/chloroform mix and once with chloroform, before precipitation of the transcript by the addition of 1/3 volume of 7.5 M ammonium acetate and incubation on ice for at least 2 hours. After recovery of the transcript by centrifugation and washing, the degree of incorporation of ^32^P-labelled uridine was measured by Cerenkov counting of the transcript and the unincorporated UTP. The Tetrahymena intervening sequence transcript was generated from the ScaI digested pT7L-21 vector [24] (ATCC 40291) using the same method. The PDGF transcript was generated by T3 RNA polymerase transcription from the Eco RI digested vector pBSPDGF-LA using an Ampliscribe T3 kit (Epicentre Technologies, Madison, WI, USA). The vector pBSPDGF-LA was generated by insertion of the Xba I/Eco RI fragment from pmetPDGF-LA [42] into a similarly digested pBluescribe vector.

### Oligonucleotide sequences

Deoxyribonucleotides are denoted by lower case letters, ribonucleotides by uppercase letters, modified nucleotides are shown in bold, **C**^**f **^= 2'-fluoro-2'-deoxycytidine, **C**^**me **^= 2' O-methylcytidine, **U**^**am **^= 2'-deoxy-2'-aminouridine, **U**^**me **^= 2' O-methyluridine, **ps **= phosphorothioate linkage. S-30, UAA CUU AUC AAG GUC* AAG AAC AUG AUG UUc, * denotes site of cleavage; Mrz-8/8, atgttctt CUGAUGA gttttc GAAAC cttgat; Mrz-12/12, catcatgttctt CUGAUGA gttttc GAAAC cttgataagt; Mz-12/12 catcatgttctt CUGAUGA gtttt GAAAC cttgataagt; Rz-12/12, catcatgttctt CUGAUGA GUCC UUUU GGAC GAAAC cttgataagt; Rz-6/12, gttctt CUGAUGA GUCC UUUU GGAC GAAAC cttgataagt; Mrz-12/12-A (= Mrz-12/12), catcatgttctt CUGAUGA gttttc GAAAC cttgataagt; Mrz-12/12-B, catcatgttctt **C**^**f**^**U**^**am**^GA**U**^**am**^GA gttttc GAAA**C**^**f **^cttgataagt; Mrz-12/12-C, catcatgttctt C**U**^**am**^GA**U**^**am**^GA gttttc GAAAC cttgataagt; Mrz-12/12-D, catcatgttctt C**U**^**am**^GAUGA gttttc GAAAC cttgataagt; Mrz-12/12-E, catcatgttctt CUGA**U**^**am**^GA gttttc GAAAC cttgataagt; Mrz-12/12-F, catcatgttctt CtGAUGA gttttc GAAAC cttgataagt; Mrz-12/12-G, catcatgttctt CuGAUGA gttttc GAAAC cttgataagt; Mrz-12/12-H, catcatgttctt C**U**^**me**^GAUGA gttttc GAAAC cttgataagt; Mrz-12/12-I, catcatgttctt CU**ps**GAUGA gttttc GAAAC cttgataagt; Mrz-12/12-J, catcatgttctt **C**^**me**^U**ps**GA**U**^**me**^GA gttttc GAAA**C**^**me **^cttgataagt**ps**t**ps**t; Mrz-12/12-K, catcatgttctt **C**^**me**^**U**^**am**^GA**U**^**me**^GA gttttc GAAA**C**^**me **^cttgataagt; Rz-12/12-L, catcatgttctt C**U**^**am**^GAUGA GUCC UUUU GGAC GAAAC cttgataagt; Tet Mrz, gcaatctattggtttaaa CUGAUGA gttttc GAAAC tagctaccaggtgcatg 3'; HC Mrz, 5' gtcgccacgacgac CUGAUGA gttttc GAAAC gttcccgctggt 3'; HC Rz, 5' gtcgccacgacgac CUGAUGA GGCC GAAA GGCC GAAAC gttcccgctggt 3'; HC S29, 5' ACCAGCGGGAACGUCGUCGUCGUGGCGAc 3'; PDGF Mrz, 5' CAGCUUCCUC CUGAUGA ggtaac GAAAU GCUUCUCt 3' ; PDGF S25, 5' GAAGAGAAGCAUCGAGGAAGCUGUc 3'.

### Cleavage kinetics

Cleavage kinetics were studied at 37°C, pH 7.6 and 10 mM MgCl_2 _under conditions of ribozyme excess; the substrate concentrations varied between 4 and 20 nM, and the ribozyme concentrations were between 20 nM and 10 μM. The short, synthetic substrates were labelled at their 5' ends using polynucleotide kinase (Roche Molecular Biochemicals) and γ-^32^P ATP. The long, transcribed substrates were uniformly labelled by including α-^32^P UTP in the transcription reaction. The ribozymes and ^32^P-labelled substrates were mixed together in 20 μL of 50 mM Tris buffer and heated to 85°C for two minutes, then incubated at 37°C for 2 minutes. The tube containing the mix was centrifuged, and 2 μL was removed and put into 4 μL quenching solution which contained 90% formamide, 20 mM EDTA and 0.01 % xylene cyanol and bromophenol blue. The reaction was initiated by the addition of 2 μL of 100 mM MgCl_2_, and 2 μL samples were removed at various times and quenched as described above. The reaction products were separated using 15% denaturing polyacrylamide gels (containing 7 M urea), and then imaged using a Molecular Dynamics PhosphorImager. At each time-point, the amount of the substrate cleaved was calculated and plotted versus time. The data were fitted by a least-squares method using the program MacCurveFit [43], to an equation of the form: P_t _= P_∞_-(exp(-k_obs_t)P_Δ_) where P_t _is the amount of product at time t, P_∞ _is amount of product generated in the exponential phase of the reaction, k_obs _is the first-order rate constant for the reaction, and P_Δ _is the difference in the amount of product at t = 0 and P_∞_. Some reactions resulted in biphasic kinetics in which the first-order phase described above was followed by a slower step which was accommodated adequately in the curve-fitting process by the addition of a linear term, P_t _= {P_∞_-(exp(-k_obs_t)P_Δ_)} + d*t. Apparent dissociation constants "Kd" and the maximal first-order rate constants k_max _were determined from the plot of k_obs _versus ribozyme concentration according to the simple binding isotherm: k_max _= (k_obs _* [Rz])/("K_d_" + [Rz]) by least-squares fitting using the program MacCurveFit.

### Serum stability

Ribozymes labelled at their 5' end with ^32^P phosphate were dissolved in RPMI medium (Gibco) at 2 μM and the reactions were initiated by adding human serum (pooled human serum, Red Cross Blood Bank, Sydney, NSW), to a final concentration of 10%. A 2 μL sample was removed immediately and the remainder incubated at 37°C with samples removed at the times indicated in Figures 2,3,4. The samples were quenched by addition to 4 μL of 90% formamide containing 20 mM EDTA and 0.01% bromophenol blue and xylene cyanol. The products of nuclease digestion in each sample were separated on 15% polyacrylamide gels containing 7 M urea and imaged using a Molecular Dynamics PhosphorImager.

## Authors' contributions

The authors jointly conceived and designed this study. PH synthesised the ribozymes and performed the kinetic and stability analyses. All authors read and approved the final manuscript.

## References

[B1] Buzayan JM, Gerlach WL, Bruening G (1986). Non-Enzymatic cleavage and ligation of RNAs complementary to a plant virus satellite RNA. Nature.

[B2] Hutchins CJ, Rathjen PD, Forster AC, Symons RH (1986). Self-cleavage of plus and minus RNA transcripts of avocado sunblotch viroid. Nucleic Acids Res.

[B3] Symons RH (1992). Small catalytic RNAs. Ann Rev Biochem.

[B4] Uhlenbeck OC (1987). A small catalytic oligoribonucleotide. Nature.

[B5] Haseloff J, Gerlach WL (1988). Simple RNA enzymes with new and highly specific endoribonuclease activitie. Nature.

[B6] Perriman R, Delves A, Gerlach WL (1992). Extended target-site specificity for a hammerhead ribozyme. Gene.

[B7] Shimayama T, Nishikawa S, Taira K (1995). Generality of the NUX rule: kinetic analysis of the results of systematic mutations in the trinucleotide at the cleavage site of hammerhead ribozymes. Biochemistry.

[B8] Zoumadakis M, Tabler M (1995). Comparative analysis of cleavage rates after systematic permutation of the NUX consensus target motif for hammerhead ribozymes. Nucleic Acids Res.

[B9] Yang JH, Usman N, Chartrand P, Cedergren R (1992). Minimum ribonucleotide requirement for catalysis by the RNA hammerhead domain. Biochemistry.

[B10] Hertel KJ, Pardi A, Uhlenbeck OC, Koizumi M, Ohtsuka E, Uesugi S, Cedergren R, Eckstein F, Gerlach WL, Hodgson R, Symons RH (1992). Numbering system for the hammerhead. Nucleic Acids Res.

[B11] Shimayama T, Nishikawa F, Nishikawa S, Taira K (1993). Nuclease-resistant chimeric ribozymes containing deoxyribonucleotides and phosphorothioate linkages. Nucleic Acids Res.

[B12] Paolella G, Sproat BS, Lamond AI (1992). Nuclease resistant ribozymes with high catalytic activity. EMBO J.

[B13] Pieken WA, Olsen DB, Benseler F, Aurup H, Eckstein F (1991). Kinetic characterisation of ribonuclease-resistant 2'-modified hammerhead ribozymes. Science.

[B14] Heidenreich O, Benseler F, Fahrenholz A, Eckstein F (1994). High activity and stability of hammerhead ribozymes containing 2'-modified pyrimidine nucleosides and phosphorothioates. J Biol Chem.

[B15] Scherr M, Klebba C, Haner R, Ganser A, Engels JW (1997). Synthesis and properties of hammerhead ribozymes stabilised against nucleases by different 2'-modifications: methoxyethoxy-, fluoro- and amino groups. Bioorg Med Chem Lett.

[B16] Beigelman L, McSwiggen JA, Draper KG, Gonzalez C, Jensen K, Karpeisky AM, Modak AS, Matulic-Adamic J, DiRenzo AB, Haeberli P, Sweeder D, Tracz D, Grimm S, Wincott FE, Thackray VG, Usman N (1995). Chemical modification of hammerhead ribozymes. J Biol Chem.

[B17] McCall MJ, Hendry P, Jennings PA (1992). Minimal sequence requirements for ribozyme activity. Proc Natl Acad Sci U S A.

[B18] McCall MJ, Hendry P, Lockett TJ (1997). Minimized hammerhead ribozymes. Methods Mol Biol.

[B19] McCall MJ, Hendry P, Mir AA, Conaty J, Brown G, Lockett TJ (2000). Small, efficient hammerhead ribozymes. Mol Biotechnol.

[B20] Hendry P, McCall MJ (1996). Unexpected anisotropy in substrate cleavage rates by asymmetric hammerhead ribozymes. Nucleic Acids Res.

[B21] Hendry P, McCall MJ, Lockett TJ (1997). Design of hybridizing arms in hammerhead ribozymes. Methods Mol Biol.

[B22] Flores MV, O'Sullivan WJ, Stewart TS (1994). Characterisation of the carbamoyl phosphate synthetase gene from Plasmodium falciparum. Mol Biochem Parasitol.

[B23] Flores MVC, Atkins D, Wade D, O'Sullivan WJ, Stewart TS (1997). Inhibition of *Plasmodium falciparum *proliferation in vitro by ribozymes. J Biol Chem.

[B24] Herschlag D, Piccirilli JA, Cech TR (1991). Ribozyme-catalyzed and nonenzymatic reactions of phosphate diesters: rate effects upon substitution of sulfur for a nonbridging phosphoryl oxygen atom. Biochemistry.

[B25] Clouet-d'Orval B, Uhlenbeck OC (1997). Hammerhead ribozymes with a faster cleavage rate. Biochemistry.

[B26] Bassi GS, Mollegaard NE, Murchie AI, Lilley DM (1999). RNA folding and misfolding of the hammerhead ribozyme. Biochemistry.

[B27] Blount KF, Grover NL, Mockler V, Beigelman L, Uhlenbeck OC (2002). Steric interference modification of the hammerhead ribozyme. Chem Biol.

[B28] Hammann C, Lilley DMJ (2002). Folding and activity of the hammerhead ribozyme. ChemBioChem.

[B29] Borda EJ, Markley JC, Sigurdsson STh (2003). Zinc-dependent cleavage in the catalytic core of the hammerhead ribozyme: evidence for a pH-dependent conformational change. Nucl Acids Res.

[B30] Murray JB, Dunham CM, Scott WG (2002). A pH-dependent conformational change, rather than the chemical step, appears to be rate-limiting in the hammerhead ribozyme cleavage reaction. J Mol Biol.

[B31] Dunham CM, Murray JB, Scott WG (2003). A helical twist-induced conformational switch activates cleavage in the hammerhead ribozyme. J Mol Biol.

[B32] Scott WG, Finch JT, Klug A (1995). The crystal structure of an all-RNA hammerhead ribozyme: a proposed mechanism for RNA catalytic cleavage. Cell.

[B33] Pley HW, Flaherty KM, McKay DB (1994). Three-dimensional structure of a hammerhead ribozyme. Nature.

[B34] Hormes R, Sczakiel G (2002). The size of hammerhead ribozyme is related to cleavage kinetics: the role of substrate length. Biochimie.

[B35] Knoll R, Bald R, Furste JP (1997). Complete identification of nonbridging phosphate oxygens involved in hammerhead cleavage. RNA.

[B36] Ruffner DE, Uhlenbeck OC (1990). Thiophosphate interference experiments locate phosphates important for the hammerhead RNA self-cleavage reaction. Nucl Acids Res.

[B37] Koziolkiewicz M, Owczarek A, Gendaszewska E (1999). Enzymatic assignment of diastereomeric purity of stereodefined phosphorothioate oligonucleotides. Antisense Nucleic Acid Drug Dev.

[B38] Maier M, Bleicher K, Kalthoff H, Bayer E (1995). Enzymatic degradation of various antisense oligonucleotides: monitoring and fragment identification by MECC and ES-MS. Biomed Pept Proteins Nucleic Acids.

[B39] Montgomery MK (2004). RNA interference: historical overview and significance. Methods Mol Biol.

[B40] Khvorova A, Lescoute A, Westhof E, Jayasena SD (2003). Sequence elements outside the hammerhead ribozyme catalytic core enable intracellular activity. Nature Struct Biol.

[B41] De la Pena M, Gago S, Flores R (2003). Peripheral regions of natural hammerhead ribozymes greatly increase their self-cleavage activity. EMBO J.

[B42] Kelly JL, Sanchez A, Brown GS, Chesterman CN, Sleigh MJ (1993). Accumulation of PDGF B and cell-binding forms of PDGF A in the extracellular matrix. J Cell Biol.

[B43] Kevin Raner Software. http://www.krs.com.au/products.html.

